# Sleep disturbances predict prospective declines in resident physicians’ psychological well-being

**DOI:** 10.3402/meo.v20.28530

**Published:** 2015-07-21

**Authors:** Alice A. Min, David A. Sbarra, Samuel M. Keim

**Affiliations:** 1 Department of Emergency Medicine, University of Arizona, Tucson, AZ, USA; 2 Department of Psychology, University of Arizona, Tucson, AZ, USA

**Keywords:** wellness, sleep quality, residency training

## Abstract

**Background:**

Medical residency can be a time of increased psychological stress and sleep disturbance. We examine the prospective associations between self-reported sleep quality and resident wellness across a single training year.

**Methods:**

Sixty-nine (*N*=69) resident physicians completed the Brief Resident Wellness Profile (*M*=17.66, standard deviation [SD]=3.45, range: 0–17) and the Pittsburgh Sleep Quality Index (*M*=6.22, SD=2.86, range: 12–25) at multiple occasions in a single training year. We examined the 1-month lagged effect of sleep disturbances on residents’ self-reported wellness.

**Results:**

Accounting for residents’ overall level of sleep disturbance across the entire study period, both the concurrent (within-person) within-occasion effect of sleep disturbance (*B*=−0.20, standard error [SE]=0.06, *p=*0.003, 95% confidence interval [CI]: −0.33, −0.07) and the lagged within-person effect of resident sleep disturbance (*B=*−0.15, SE=0.07, *p=*0.037, 95% CI: −0.29, −0.009) were significant predictors of decreased resident wellness. Increases in sleep disturbances are a *leading indicator* of resident wellness, predicting decreased well-being 1 month later.

**Conclusions:**

Sleep quality exerts a significant effect on self-reported resident wellness. Periodic evaluation of sleep quality may alert program leadership and the residents themselves to impending decreases in psychological well-being.

For new physicians, residency training is a time to gain knowledge in their chosen specialty and hone clinical skills. Many resident physicians, however, also experience significant psychological stress and sleep deprivation, both of which have detrimental effects on learning and clinical performance. Among residents, feelings of burnout, fatigue, depression, sleepiness, and reduced quality of life are all independently associated with an increased risk of future self-perceived major medical errors (1). Reforms in graduate medical education (e.g., duty hour restrictions) are aimed at reducing fatigue. However, the issue of resident psychological distress persists. A method to help residency program leadership measure and intervene on fatigue and decreasing wellness may identify impending issues before clinical performance and personal well-being are affected. In a study spanning 9 months, we evaluated whether within-person variability in residents’ sleep quality predicted changes in their overall self-reported wellness over time.

## Methods

**Fig. 1:**
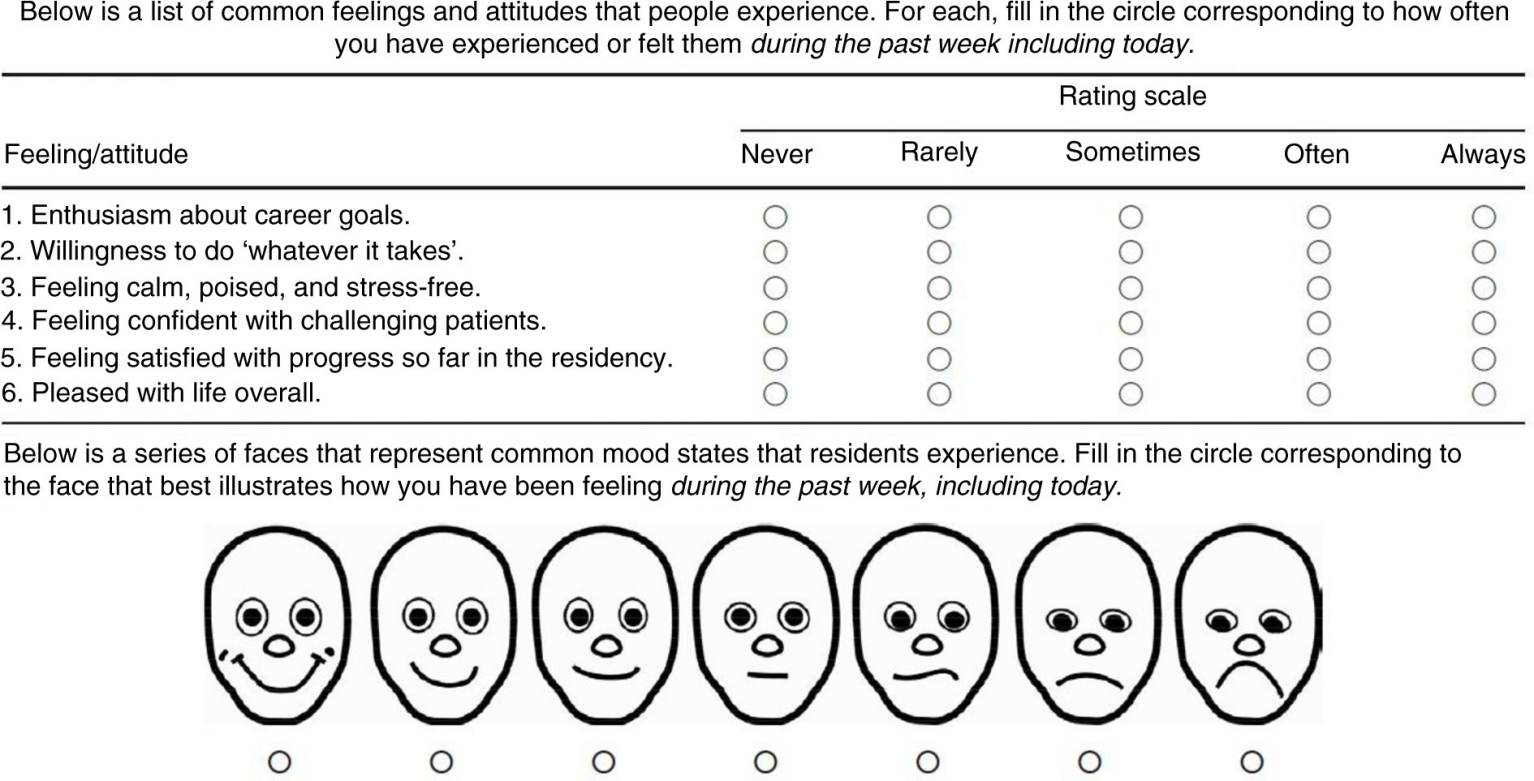
Brief Resident Wellness Profile.

At a large teaching hospital, 69 (*N*=69) resident physicians at variable post-graduate years from a range of specialties completed the standardized, Brief Resident Wellness Profile (2) (BRWP; *M*=17.66, standard deviation [SD]=3.45, range: 0–17) and the Pittsburgh Sleep Quality Index (3) (PSQI; *M*=6.22, SD=2.86, range: 12–25) at multiple occasions beginning in September of a single training year (range 1–9 completed reports, 182 total observations). The BRWP asked participants to rate items about their general levels of psychological stress, satisfaction with their residency and life in general, and enthusiasm for their career goals; the PSQI is a subjective measure of sleep disturbance, which asks participants to rate the quality of their sleep in the prior 30 days, including how often they have had trouble falling asleep, how often they have used sleep medication, and how often they have had trouble staying awake while performing activities of daily living. Both measures provide reliable and valid information in their respective domains (2, 3), and the PSQI is a widely used self-reported assessment of subjective sleep disturbance (4). The study was approved by the local institutional review board (Fig. 1).

The trajectory of self-reported resident wellness over the entire study period was modeled using a linear mixed model (5) that treated PSQI scores at each occasion as a time-varying predictor. PSQI scores were person-centered against each physician's average sleep disturbance scores across the entire assessment period. We examined the 1-month lagged effect of within-person sleep disturbances on residents’ self-reported wellness accounting for concurrent sleep disturbance and, to control for between-person effects, residents’ average sleep disturbance across the entire assessment period (as a level-2 covariate in the mixed model).

## Results

A basic multilevel growth model revealed no systematic declines (i.e., linear change) in resident wellness across the training year (*B*=−0.04, standard error [SE]=0.07*, p*=0.51, 95% confidence interval [CI]: −0.19, 0.09). Accounting for residents’ overall level of sleep disturbance across the entire study period, both the concurrent (within-person) within-occasion effect of sleep disturbance (*B*=−0.20, SE=0.06, *p=*0.003, 95% CI: −0.33, −0.07) and the lagged within-person effect of resident sleep disturbance (*B=*−0.15, SE=0.07, *p=*0.037, 95% CI: −0.29, −0.009) were significant predictors of decreased resident wellness. Increases in sleep disturbances are a *leading indicator* of resident wellness, predicting decreased subjective well-being 1 month later (accounting for concurrent sleep problems). In a reverse model, with sleep disturbances as the outcome, lagged wellness does not predict future sleep disturbances, and these effects remained stable after accounting for residents’ medical specialty.

In calibrated terms, residents reporting 2 months of sleep disturbance that fell, for example, at 1 SD above their own average sleep disturbance reported experiencing 0.22 of a SD decrease in their overall wellness scores. For residents who reported sleeping well in general (at or below the mean on the PSQI across the entire study period), this change does not have a meaningful effect on their overall wellness (i.e., the change predicts variability entirely within the high range of resident wellness). For residents who reported high levels of sleep disturbance across the study period (at or above 1 SD of the mean on PSQI scores across the entire study period), 2 months of sleep disturbances (relative to a person's own mean scores) have the effect of shifting wellness scores to the 30th percentile or below. Thus, from an intervention standpoint, residents at the highest risk for clinically meaningful decreases in their psychological well-being appear to be those who reported a worsening of already poor sleep.

## Discussion

Fatigue and wellness clearly impact residents’ training and clinical performance. Papp et al. (6) found that sleep loss and fatigue had major impact on all areas of residents’ lives. Learning and cognition were affected as fatigue caused a lack of motivation and difficulty with higher order thinking. Job performance, including less professional communication with staff, lack of empathy for patient and families, resistance to performing tasks, and perceived medical errors, also was impacted by sleep deprivation. Residents’ personal lives, specifically physical and emotional health and interpersonal relationships, suffered from fatigue and sleep loss as well (6).

Our findings suggest that sleep quality exerts a significant *and* meaningful lagged effect on self-reported resident wellness. This finding was robust over a variety of specialties and all levels of training. Program directors may find this useful to predict and prevent declines in the wellness of their residents. It has been shown that people experiencing persistent sleep problems are 3.5 times more likely to experience a subsequent depressive episode than people without sleep problems (7). Periodic evaluation of the residents’ sleep quality may alert program leadership and the residents themselves to impending decreases in psychological well-being and provide an opportunity for intervention before negative effects on their learning, job performance, and personal lives are significant.

The findings from this study should be considered in light of its limitations. The overall sample size was relatively small, but the repeated assessments bolster the statistical power to detect significant effects in the prospective mixed model. In addition, the measurement of wellness and sleep disturbance was both subjective, and it would be beneficial to have multiple methods of assessment. Future studies should measure the effect of wellness and sleep quality on clinical performance as evaluated by supervising faculty or In-Training Examination scores. Another limitation of this study is the effect confounding variables have on wellness and sleep quality, including work hours and personal life stressors. We attempted to obtain wellness scores from domestic partners of residents; however, the responses were limited. A critical goal for future research on sleep and wellness will be to replicate the lagged effect and rule-out competing explanations due to confounding variables.

## Conclusion

Future research to further evaluate the validity and utility of tools to predict issues in decreased wellness is needed. Our findings suggest that poor sleep quality is an indicator of future (self-reported) decreases in resident wellness, which suggests that poor sleep may presage declines in psychological well-being.

## References

[1] West CP, Tan AD, Habermann TM, Sloan JA, Shanafelt TD (2009). Association of resident fatigue and distress with perceived medical errors. JAMA.

[2] Keim SM, Mays MZ, Williams JM, Serido J, Harris RB (2006). Measuring wellness among resident physicians. Med Teach.

[3] Buyssee DJ, Reynolds CF, Monk TH, Berman SR, Kupfer DJ (1989). The Pittsburgh Sleep Quality Index: a new instrument for psychiatric practice and research. Psychiatry Res.

[4] Hayashino Y, Yamazaki S, Takegami M, Nakayama T, Sokejima S, Fukuhara S (2010). Association between number of comorbid conditions, depression, and sleep quality using the Pittsburgh Sleep Quality Index: results from a population-based survey. Sleep Med.

[5] Singer JD, Willett JB (2003). Applied longitudinal data analysis: modeling change and event occurrence.

[6] Papp KK, Stoller EP, Sage P, Aikens JE, Owens J, Avidan A (2004). The effects of sleep loss and fatigue on resident-physicians: a multi-institutional mixed-method study. Acad Med.

[7] Johnson EO, Roth T, Breslau N (2006). The association of insomnia with anxiety disorders and depression: exploration of the direction of risk. J Psychiatr Res.

